# Paramedic Pain Management Practice with Introduction of a Non-opiate Treatment Protocol

**DOI:** 10.5811/westjem.2020.6.47032

**Published:** 2020-08-21

**Authors:** Laurel O’Connor, Julianne Dugas, Jeffrey Brady, Andrew Kamilaris, Steven K. Shiba, Ricky C. Kue, John P. Broach

**Affiliations:** * University of Massachusetts Medical School, Department of Emergency Medicine, Worcester, Massachusetts; † Boston Medical Center, Department of Emergency Medicine, Boston, Massachusetts; ‡ Charles E. Schmidt College of Medicine at Florida Atlantic University, Boca Raton, Florida; § South Shore Health, Department of Emergency Medicine, South Weymouth, Massachusetts

## Abstract

**Introduction:**

There is concern about the initiation of opiates in healthcare settings due to the risk of future misuse. Although opiate medications have historically been at the core of prehospital pain management, several states are introducing non-opiate alternatives to prehospital care. Prior studies suggest that non-opiate analgesics are non-inferior to opiates for many acute complaints, yet there is little literature describing practice patterns of pain management in prehospital care. Our goal was to describe the practice patterns and attitudes of paramedics toward pain management after the introduction of non-opiates to a statewide protocol.

**Methods:**

This study was two-armed. The first arm employed a pre/post retrospective chart review model examining medication administrations reported to the Massachusetts Ambulance Trip Information System between January 1, 2017–December 31, 2018. We abstracted instances of opiate and non-opiate utilizations along with patients’ clinical course. The second arm consisted of a survey administered to paramedics one year after implementation of non-opiates in the state protocol, which used binary questions and Likert scales to describe beliefs pertaining to prehospital analgesia.

**Results:**

Pain medications were administered in 1.6% of emergency medical services incidents in 2017 and 1.7% of incidents in 2018. The rate of opiate analgesic use was reduced by 9.4% in 2018 compared to 2017 (90.6% vs 100.0%). The absolute reduction in opiate use in 2018 was 3.6%. Women were less likely (odds ratio [OR] = 0.78, 95% confidence interval [CI], 0.69–0.89) and trauma patients were more likely to receive opiates (OR = 2.36, CI, 1.96–2.84). Mean transport times were longer in opiate administration incidents (36.97 vs 29.35 minutes, t = 17.34, p<0.0001). We surveyed 100 paramedics (mean age 41.98, 84% male). Compositely, 85% of paramedics planned to use non-opiates and 35% reported having done so. Participants planning to use non-opiates were younger and less experienced. Participants indicated that concern about adverse effects, efficacy, and time to effect impacted their practice patterns.

**Conclusion:**

The introduction of non-opiate pain medication to state protocols led to reduced opiate administration. Men and trauma patients were more likely to receive opiates. Paramedics reported enthusiasm for non-opiate medications. Beliefs about non-opioid analgesics pertaining to adverse effects, onset time, and efficacy may influence their utilization.

## INTRODUCTION

The management of acute pain is central to emergency care in both the hospital and prehospital settings. Pain prevalence in the prehospital setting has been reported to be between 42–53%.^1^–^2^ Both the National Association of Emergency Medical Services Physicians and the American College of Emergency Physicians have emphasized the importance of addressing the high incidence of pain reported by emergency medical services (EMS) utilizers.^3^–^4^ However, recent literature suggests that many prehospital providers undertreat prehospital pain complaints.^5^–^7^

The availability of specific types of analgesics varies by state, but traditionally opiates have been at the core of prehospital analgesia protocols. This precedent is complicated by the ongoing national opiate crisis and the concern that utilization of opiates in the acute setting may engender long-term misuse and addiction. The opioid epidemic has been prominent in the minds of healthcare providers and has had major impacts on how analgesics are dispensed.^8^ While there has been some research linking administration of opiates in the acute care setting with recurrent opiate use, this phenomenon has not been well described in the prehospital setting.^9^

In Massachusetts, acetaminophen, ketorolac, and ibuprofen were introduced to the pain management protocol for paramedics on January 1, 2018.^10^ While these medications have a favorable safety profile and do not have the addictive or sedative qualities that render opiates dangerous, they do have the potential to cause harm in some patients. Ibuprofen and ketorolac have an adverse effect profile that includes gastric bleeding, renal dysfunction, and platelet derangement; acetaminophen may contribute to hepatic toxicity.^11^ While those adverse effects are relatively uncommon, there is some concern about administering these medications in the prehospital setting in which the patient is undifferentiated and diagnostic testing is extremely limited. There is also the barrier of widespread belief that non-opiates are less efficacious than opiates for acute pain and have an unacceptable time to effect. However, there is a considerable body of literature demonstrating non-inferiority in non-opiate medication administration as compared to opiates for many conditions commonly encountered in the prehospital setting including renal colic, long bone fracture, and other minor traumatic limb injuries.^12^–^15^

While most clinicians are in agreement that prehospital providers should treat pain, there remains equipoise as to how to guide prehospital pain management. Providers must attempt to consolidate information pertaining to patients’ self-reported levels of pain, clinical characteristics, and the possible adverse effects of available analgesics, and then make an expeditious decision about which medication to administer. Despite the more widespread availability of non-opiate analgesics and their proven efficacy, there are few data available regarding practice patterns of prehospital providers or about their perceptions of prehospital pain management. This study sought to describe trends in prehospital analgesic use and providers’ attitudes toward pain management one year after the introduction of non-opiate options to the state protocol in Massachusetts.

**Population Health Research Capsule**

What do we already know about this issue?

In the midst of the opioid-use epidemic, the judicious use of opiate analgesics for acute pain is paramount to mitigate the risk of future misuse.

What was the research question?

We asked if there were changes in prehospital pain management after introduction of non-opiate analgesics.

What was the major finding of the study?

Prehospital opiate administration by paramedics was reduced when non-opiate options were available.

How does this improve population health?

The introduction of non-opiate medications to prehospital protocols enables paramedics to avoid opiates when appropriate.

## METHODS

### Setting and participants

This project used data from Advanced Life Support (ALS) EMS agencies across Massachusetts. All data for the retrospective chart review arm of the study were derived from the Massachusetts Ambulance Trip Recording System (MATRIS) for ambulance trip-sheets ranging from January 1, 2017–December 31, 2018.^8^,^16^ This standardized database contains data uploaded from 224 ALS-capable services in the state (with 97.9% reporting compliance), and it is National Emergency Medical Services Information System compliant.^16^ MATRIS is maintained by the Massachusetts Department of Public Health.

Paramedics for the perceptions survey arm were recruited from 16 departments representing urban, suburban, and rural services in Massachusetts. All participants were recruited at scheduled training and administrative meetings. Agencies included two hospital-based services, five fire-based services, and nine private services. Participants were considered eligible for inclusion if they were at least 18 years old, fluent in English, and currently nationally registered, licensed, and field-active paramedics. In total, 104 participants were approached and 100 completed the administered survey (96% enrollment rate).

### Procedure

We queried the MATRIS database for all administrations of morphine or fentanyl documented in prehospital trip-sheets in 2017 and all administrations of morphine, fentanyl, ketorolac, acetaminophen, or ibuprofen in 2018. Patients who received morphine or fentanyl comprised the “opiate cohort,” whereas patients who received ketorolac, acetaminophen and ibuprofen comprised the “non-opiate” cohort. After being implemented in the state protocol, non-opiate medications became available to all agencies simultaneously. We excluded all encounters where the primary and/or secondary impression was cardiac arrest or obvious death. Encounters where neither the primary impression nor secondary impression contained a pain complaint (eg, respiratory distress, respiratory arrest, etc.) were excluded in an effort to eliminate instances in which opiates were used for sedation and not as analgesics. At the time of this project, ketamine was not available for prehospital analgesia; it was used only for induction in medication-assisted intubation or for agitated delirium. Encounters where only ketamine was administered were excluded. We placed patients who received both opiate and non-opiate medications in the opiate cohort. Records in which data was incompatible with MATRIS parameters or obviously erroneous were excluded. We included encounters for which there was no primary or secondary impression listed but the dispatch chief complaint was pain related. The inclusion decision-making parameters are depicted in Figure.

For the survey portion of the project, participants were asked to complete an anonymous, 10-minute pencil-and-paper survey. The survey was conducted in the spring following the first full year of the implementation of non-opiate medications in the state protocol. No additional discussion or education regarding prehospital pain management occurred in the setting of the survey administration. Data were collected and managed using REDCap (Research Electronic Data Capture) tools (V9.1.0) hosted by the primary study site. REDCap is a secure, web-based software platform designed to support data capture for research studies.^17^–^18^ The institutional review boards at all participating institutions, as well as the Massachusetts Department of Public Health, approved this project.

### Measures

Parameters extracted from MATRIS for the data arm of this study included medication administered, dispatch complaint, subject age, gender, initial systolic blood pressure (SBP), initial heart rate (HR), transport time, primary and secondary impression, the EMS agency providing service, and the location of the EMS call. The perceptions survey collected demographic information from participants and then utilized a series of binary question and Likert scales to assess subjects’ attitudes about the benefits and barriers to using opiate and non-opiate medications for prehospital analgesia. The full language of the survey is depicted in supplemental Table 2.

### Analysis

For the MATRIS data, we calculated descriptive statistics on all measures for the three cohorts (2017 opiate patients, 2018 opiate patients, and 2018 non-opiate patients). Comparative statistics were performed for all measured patient factors including age, gender, mean initial SBP, and mean initial HR. We completed all statistical computations using SAS v9.4 (SAS Institute, Cary, NC). P-values were obtained using Welch-Satterthwaite t-testing for continuous variables such as age, SBP and HR, and chi-square testing of independence for categorical variables. We used Pearson correlation values to assess the association between county median income and the proportion of medication administration incidents involving opiates.

For the survey arm, we calculated descriptive data for all participants, and subsequently for the subgroups of participants that planned to administer non-opiates and those that did not. P-values were calculated using chi-square testing for binary variables and Wilcoxon rank-sum testing for continuous variables. The Likert scale data for perceptions data were reported descriptively for all three cohorts (all participants, participants planning to use non-opiates and participants not planning to use non-opiates). Finally, planning to use non-opiates was analyzed by demographics and Likert scale responses, using chi-square tests of independence for categorical variables and Pearson correlations for continuous variables.

## RESULTS

### MATRIS Data

Descriptive data for all chart review subjects is summarized in Table 1. Subject demographics between the pre- and post-intervention cohorts were not significantly different. In total there were 677,364 emergent field EMS responses in 2017 and 673,561 in 2018; the rate of pain medication administration was 1.6% in 2017 and 1.7% in 2018. Total medication administrations are reported in Table 2. Overall, the rate of opiate analgesic use was reduced by 9.4% in 2018. The absolute reduction in opiate use in 2018 was 3.6% (385/10809) compared with 2017. Once non-opiate options were introduced, women were more likely than men to receive an opiate medication (OR = 0.78, 95% CI, 0.69–0.89). There were no statistically significant differences in mean age between opiate and non-opiate recipients. There were small but statistically significant differences between mean initial SBP and HR (−1.93 milligrams of mercury and +1.81 beats per minute, respectively, in the opiate cohort).

In cases where a primary impression was available, most non-opiates were administered for medical chief complaints. Patients with traumatic complaints were significantly more likely to receive opiate medications (OR = 2.36, 95% CI, 1.96–2.84). In both years, and within the opiate and non-opiate cohorts, abdominal pain was the most common clinical impression for which pain medication was administered.

The ratio of opiates to total pain medication administrations by individual EMS services in 2018 ranged from 0.39 to 1.00. In total, 120 out of 224 (54%) of reporting services administered at least one non-opiate during the post-intervention year. Three services had an opiate administration ratio less than 50%, 27 had an opiate administration rate under 75%, and 74 had opiate administration rates under 90%. When the EMS services included in the survey arm were examined separately, the proportion of opiates administered in 2018 ranged from 0.81–1.00; there was no significant deviation in their administration patterns as compared to the rest of the state. We calculated Pearson correlation between proportion of opiate administration and median county income of service location and it was not significant (R = 0.25, p = 0.41).

### Paramedics Perceptions Data

In total, we surveyed 100 participants (mean age 42 years, 95% CI, 40.19–43.77; 84% male). All participant demographics and those of cohorts planning and not planning to use non-opiates are summarized in Table 3. Participants who reported planning to use non-opiates were younger (mean age 39) and less experienced (mean 11.15 years of experience) than those who did not (41.4 years, p<0.05 and 15 years of experience, p=0.01, respectively). There was no significant difference in the pain scale number at which cohorts reported as benchmarks for administering opiate medications (p = 0.238). Paramedics with greater experience and older age were more likely to administer opiates at a lower patient-reported pain scale (R= 0.32, p < 0.05 and R = 0.27, p <0.05, respectively). Responses to Likert scale-based perceptions questions are described in supplemental Table 1. The majority of paramedics (76%) reported agreeing or strongly agreeing that there was a duty to treat pain in the prehospital setting and 90% reported believing that prehospital pain management was effective. Participants not planning to give non-opiate medications were more likely to agree that pain was difficult to assess in the prehospital setting, more likely to be concerned about the adverse effects of both opiates and non-opiates, and more likely to believe that non-opiates were not effective in managing pain and took too long to work. Participants who reported that they were planning to give non-opiate medications were more likely to be concerned that administering pain medications would change patients’ clinical presentation for providers in the ED. Concerns about drug-seeking behavior and opiate tolerance were not different between cohorts.

Few participants responded affirmatively to concerns regarding adverse effects (11%), efficacy (12%), and time to effect (21%) impacting their decision to administer non-opiates. Globally, participants also reported agreement that the non-opiate ketamine should be available for prehospital analgesia (72% agreed or strongly agreed) although there was less support for lidocaine nerve block (33% agreed or strongly agreed). There was no consensus on support for implementation of more structured protocols for selecting prehospital analgesics (26% agreed or strongly agreed; 50% disagreed or strongly disagreed).

## DISCUSSION

Analysis of one state’s data a year after the advent of non-opiate options demonstrates a modest but statistically significant absolute reduction in the use of opiates. Although more work must be done, this is cautiously encouraging; the rate of opiate administration has dropped while the rate of pain medication administration has increased slightly. Although limited demographic and clinical data are available, there are some significant patterns in how medications are administered. Trauma patients and men are more likely to receive opiate medications, and women are more likely to receive non-opiates. Possible explanations for the utilization of opiates in trauma patients include the likelihood that they have more severe or apparent pathology as opposed to the undifferentiated medical patient, a higher concern for hemorrhage, or heightened concern that a trauma patient may be an operative candidate.

Previous literature has shown a significant gender disparity in acute pain management.^2^,^19^ There are not enough data from this study to determine whether the biases that have created this discrepancy factor into prehospital pain management; however, the demonstration of gender inequality in medication administration is consistent with known inequalities. Clinical parameters such as blood pressure, heart rate, and transport time do not have clinically significant differences in values with regard to the chosen analgesic.

The state’s slow incorporation of non-opiate medications may be related to unfamiliarity and some initial discomfort with adverse-effect profiles. There may also be some uncertainty as to the appropriate use of a non-opiate vs an opiate for varying levels of reported pain and degree of pathology. The overall proportion of EMS patients who receive pain medication is very low – less than 2% per year – which brings into question whether the introduction of prehospital opiate medications is a significant contributor to later opiate misuse. Although more research is needed and the use of non-opiate medications should be encouraged when appropriate, it may be that prehospital pain management is still largely inadequate and that targeting prehospital opiate use may not be the most fruitful use of resources for misuse prevention.

### Perceptions Data

Prehospital providers largely reported believing that pain management was part of their duty in the prehospital setting; however, there was controversy among respondents regarding gauging pain levels. While many prehospital providers employ the common 0–10 pain scale, there is no strict protocol requirement correlating a certain number with choice of analgesic and there was considerable range in the numbers that providers reporting being their “cut-off” for deciding to administer an opiate medication (2–10, mean 7). Notably, the majority of respondents did report a difference in their threshold to initiate an opiate vs a non-opiate with a higher number correlating with initiating an opiate. This suggests that providers are individually using an internal decision-making framework that involves stratifying pain medication choice to the level of pain reported by patients. Methods of assessing pain level and correlating this with a particular analgesic are beyond the scope of this study, but this variety demonstrates a lack of standardization in pain management and suggests that there is significant variation among providers.

Globally, apprehension about the possible adverse effects of the non-opiates was of lesser concern to the surveyed prehospital providers. Some providers expressed a concern for giving non-steroidal medications to patients who may require operative management or patients who are suspected to have internal hemorrhage. The literature, however, largely refutes the concern that one-time use leads to significant hemorrhagic complications.^20^–^21^ The other concerns significant to providers with regard to non-opiate use include the belief that non-opiates are not as efficacious as opiates and that they take too long to work. While most providers agree that there are some conditions for which an opiate medication would be considered more appropriate, there is conclusive evidence that there are many conditions common to EMS where non-opiate medications are equally efficacious with regard to both patient safety and satisfaction and therein might be considered more appropriate for use.^15^

### The Advent of Non-Opiate Options

There are additional practical considerations in the use of non-opiates in EMS. There is value in initiating non-opiate pain management immediately rather than delaying administration of the same medication after a patient’s in-hospital evaluation. A patient who has not been administered an opiate medication may be able to have a shorter ED course because there is less concern about sedation, and if applicable, he or she would be able to operate machinery and return to activity sooner. Patients who have adequate pain management with a non-opiate in the field are less likely to expect opiate-based management in the ED whereas a patient who immediately receives opiate may be more likely to expect the same in the ED, even if the diagnosis is not one that would normally require opiate medication. Finally, non-opiate medications permit patients who cannot receive opiates to attain pain management in the field. In Massachusetts, among other states, patients with a history of substance use disorder or other reasons not to receive opiate medication have access to a voluntary “non-opioid directive form,” which signals to providers that they must receive alternative medications; having a robust arsenal of other options increases the feasibility and desirability of this directive.^22^

### Future Work and Study

Future studies may seek to describe whether there is an association between prehospital opiate use, ED opiate use, and long-term opiate use. More surveillance of prehospital practice patterns as providers become more familiar with non-opiate analgesics is needed. There have been studies demonstrating a reduction in overall opiate utilization in the acute care setting when non-opiate pain management options are made first line in pain management protocol; an extension of this type of trial to the prehospital setting is an important avenue of exploration.^23^,–^24^ Finally, there are a number of additional pain management adjuncts including ketamine, lidocaine nerve blocks, and nitrous oxide that have not been universally implemented in the prehospital setting; these may be validated as highly efficacious, prehospital pain management options.

## LIMITATIONS

Both arms of this study had multiple limitations. The data collected were in a single state with its own protocols and therefore have limited generalizability to the rest of the country. There were relatively limited demographic and clinical data available for patient subjects, and the doses of the medications administered were not available. ED data and final diagnoses were not available for subjects. As with the introduction of many protocols, there may be lag time between the implementation phase of the intervention and the prevalence of provider use of the intervention, so it is possible that data from coming years will yield a more representative depiction of pain management practice patterns. The survey arm of the study was limited to 100 providers and may not be representative of all licensed paramedics in the state. The proportion of each agency that participated in the survey was not recorded due to concerns about anonymity, and therefore one agency may have been relatively over-represented. Subjects were recruited as a convenience sample, which may have biased the results. Finally, a Hawthorne effect may have created bias given the current cultural environment pertaining to opiates.

## CONCLUSION

Non-opiate medications have been modestly incorporated into one state’s practice a year after introduction. Limited data are available on providers’ patterns of pain management, but there are some trends that may inform future educational opportunities for the medical director. Paramedics largely report enthusiasm for the non-opiate analgesic. The prehospital setting would benefit from more literature describing the efficacy of prehospital pain management and its contribution to the clinical course of acute care patients.

## Supplementary Information



## Figures and Tables

**Figure:**
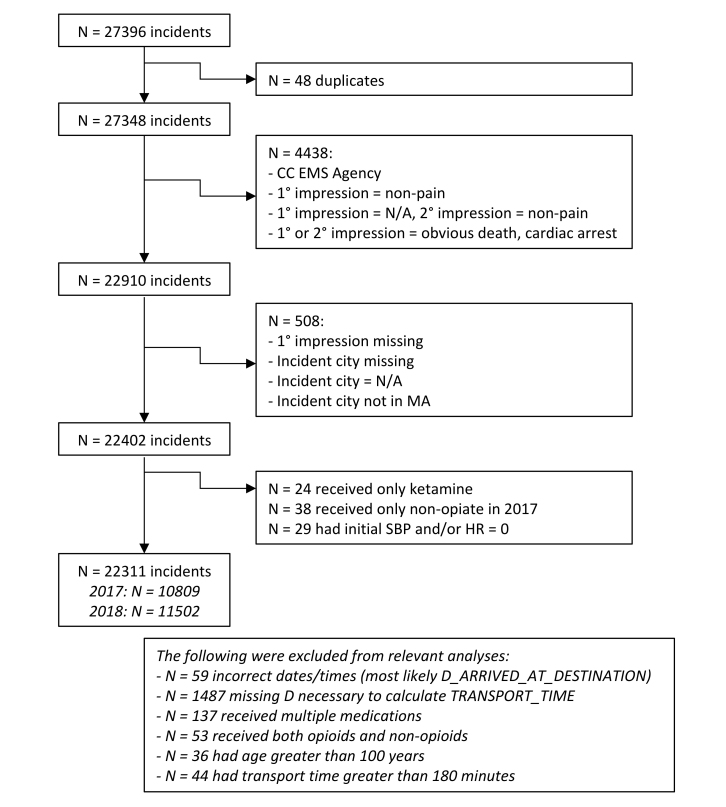
Inclusion decision-making parameters for paramedic administration of opiate and non-opiate medications. *EMS*, emergency medical services; *SBP*, systolic blood pressure; *HR*, heart rate.

**Table 1:** MATRIS+ patient demographics.

	2017 (n = 10809)		2018 (n = 11502)		
		
	Opiate§	Non-opiate*	Opiate§	Non-opiate*	P-value
Age, mean (SD)	53.7 (22.193)	N/A	54.01 (22.34)	55.31 (21.31)	0.218
Gender, n (%)					
Male	5237 (48.6)	N/A	5027 (48.4)	454 (42.3)	0.266
Female	5537 (51.4)	N/A	5352 (51.6)	619 (57.7)	
Mean transport time minutes (SD)	36.12 (18.89)	N/A	36.98 (20.33)	29.35 (29.35)	
Mean initial SBP mm hg, (SD)	143.46 (26.91)	N/A	143.75 (26.55)	145.68 (25.66)	
Mean initial HR bpm, (SD)	88.22 (19.24)	N/A	88.38 (19.42)	86.64 (18.49)	

+ Massachusetts Ambulance Trip Recording System

§ Opiate category includes morphine and fentanyl.

* Non-opiate category includes ibuprofen, acetaminophen and ketorolac.

*SD*, standard deviation; *SBP;* systolic blood pressure; *mm hg*, millimeters of mercury; *bpm*, beats per minute.

**Table 2:** Medication administration by year.

Annual totals						
	2017	2018				P-value
					
Total EMS calls	677,364	673,561				
Total administrations	10,809	11,502				<0.001
Opiates	10,809 (100%, 95% CI, 99.96–100)	10,424 (90.63%, 95% CI, 90.08–91.15)				0.002
Non-opiates	N/A	1,078				N/A

Medication Administration Demographics 2018						

	Opiate§, n	Non-opiate*, n	OR	T	Δ mean (95% CI)	P-value
	
Female	5,352	619	0.781, 95% CI 0.688–0.887			< 0.001
Male	5,027	454				
Mean age (SD)	54.01 (22.34)	55.31 (21.31)		T= 1.90		0.058
Mean transport time (minutes, SD)	36.98 (20.33)	29.35 (29.35)			−7.631 (−8.494,−6.768)	< 0.001
Mean initial SBP (mm Hg, SD)	143.75 (26.55)	29.35 (29.35)			−1.930 (0.201,3.659)	0.029
Mean initial HR, bpm (SD)	88.38 (19.42)	86.64 (18.49)			1.808 (−3.004,−0.613)	0.003

§ Opiate category includes morphine and fentanyl.

* Non-opiate category includes ibuprofen, acetaminophen and ketorolac.

*EMS*, emergency medical services; *OR*, odds ratio; *SD*, standard deviation; *SBP*, systolic blood pressure; *mm hg*, millimeters of mercury; *HR*, heart rate; *bpm*, beats per minute; *CI*, confidence interval.

**Table 3:** Survey respondent demographics.

	All Subjects	Plan to give non-opiates	Do not plan to give non-opiates	P-value
Age				
Mean	41.98	38.91	41.4	< 0.01
Median	37	36	42	
Range	24,62	24,62	26,53	
Gender, n (%)				
Male	84 (84)	69 (82)	14 (93)	0.45
Female	16 (16)	15 (18)	1 (7)	
Years of Experience				
Mean	11.73	11.15	15	0.01
Median	10	10	12	
Range	1,34	1,34	1,26	
Have given fentanyl to a patient, n (%)				
Yes	95 (95)	80 (94.1)	15 (100)	0.954
No	5 (5)	5 (5.9)	0 (0)	
Have given non-opiate to a patient, n (%)				
Yes	35 (35)	35 (41.2)	0 (0)	N/A
No	65 (65)	50 (58.8)	15 (100)	
Plan to give acetaminophen, ketorolac or ibuprofen, n (%)				
Yes	85 (85)	85 (100)	0 (0)	N/A
No	15 (15)	0 (0)	15 (100)	
Pain scale at which opiate given, n (%)				
Mean	7.02	6.94	7.5	0.238
Median	7	7	7.5	
Range	2,10	2,10	5,10	
Pain scale at which non-opiate given, n (%)				
Mean	4.21	4.21	N/A	N/A
Median	4	4	N/A	
Range	1,10	1,10		
