# Supplementary material for: Paramedic Pain Management Practice with Introduction of a Non-opiate Treatment Protocol
Source: West J Emerg Med. 2020 Aug 21;21(5):1234–41. doi: 10.5811/westjem.2020.6.47032 (PMC7514409; doi:10.5811/westjem.2020.6.47032)
Supplement: Supplementary file 1 [file wjem-21-1234-s001.docx]

| **Supplemental Table 1.** Perceptions Data. | | | |  | |  |  |  |  | |  |  | | |  |  |
| --- | --- | --- | --- | --- | --- | --- | --- | --- | --- | --- | --- | --- | --- | --- | --- | --- |
|  | **All Subjects** | | | **No Plan to give non-opiates** | | | | | | **Plan to give non-opiates** | | | | | |  |
|  | **Strongly Agree or Agree N(%)** | **Neutral N(%)** | **Disagree or Strongly Disagree N(%)** | **Strongly Agree or Agree N(%)** | **Neutral N(%)** | | **Disagree or Strongly Disagree N(%)** | | | **Strongly Agree or Agree N(%)** | | | **Neutral N(%)** | **Disagree or Strongly Disagree N(%)** | | **p** |
| **It is difficult to accurately gauge pain levels in the prehospital setting** | **33(34)** | **21 (22)** | **43(44)** | **10(66.7)** | **2 (13.3)** | | **3 (20)** | | | **23(28.0)** | | | **19(23.2)** | **40(48.8)** | | **0.007** |
| I do not give pain medication if the patient doesn't have an "obvious" medical problem/injury | **49(49)** | 19(19) | 32(32) | 10(66.7) | 1(6.7) | | 4, 26.7%) | | | 39(47.6) | | | 18(21.2) | 28(34.1) | | 0.357 |
| I have a duty to provide pain relief in the prehospital setting | 76(76) | 18(18) | 6(6) | 11(73.3) | 4(26.7) | | 0(0) | | | 65(79.3) | | | 14(16.5) | 6(7.3) | | 0.989 |
| Pain relief in the prehospital setting is effective: | 90(90) | 4(4) | 6(6) | 10(66.7) | 3(20.0) | | 2(13.3) | | | 78(94.0) | | | 1(1.2) | 4(4.81) | | 0.119 |
| The goal of pain management in the prehospital setting is to eliminate all pain | 13(13) | 10(10) | 77(77) | 4(26.7) | 2(13.3) | | 9(60.) | | | 9(10.6) | | | 8(9.4) | 68(81.9) | | 0.0703 |
| The goal of pain management is the prehospital setting is to "take the edge off" | 68(68) | 17(17) | 15(15) | 9(60.0) | 2(13.3) | | 4(36.7) | | | 59(69.4) | | | 15(17.6) | 11(12.9) | | 0.195 |
| I hesitate to give pain medication to patients who I suspect have a history of opiate abuse because I am worried that they are "drug seeking" | 53(53) | 22(22) | 25(25) | 7(46.7) | 1(6.7) | | 7(46.7) | | | 46(54.1) | | | 21(24.7) | 18(21.2) | | 0.112 |
| I hesitate to give pain medications to patients who I suspect have a history of opiate abuse because I am worried I won't be able to overcome their tolerance to pain: | 11(11) | 26(26) | 63(63) | 1(6.7) | 0(0) | | 14(93.3) | | | 49(57.6) | | | 26 (30.6) | 10(11.7) | | < 0.001 |
| **I hesitate to give pain medication to patients because I might change their clinical presentation for the emergency department** | **17(17)** | **12(12)** | **71(71)** | **2(13.3)** | **2(13.3)** | | **11(73.3)** | | | **60(70.6)** | | | **10(11.8)** | **15(17.6)** | | < 0.001 |
| My transport time impacts my decision to give pain medicine: | 29(29) | 12(12) | 57(57) | 5(33.3) | 0(0) | | 10(66.7) | | | 24(28.9) | | | 12(14.5) | 47(55.3) | | 0.972 |
| **I am hesitant to give opiate pain medicine because I am worried about the unwanted side effects:** | **6(6)** | **7(7)** | **87(87)** | **4(26.7)** | **0(0)** | | **11(73.3)** | | | **4(4.7)** | | | **6(7.1)** | **75(88.2)** | | **0.005** |
| **I am hesitant to give non-opiate pain medicine because I am worried about the unwanted side effects:** | **11(11)** | **6(6)** | **83(83)** | **4(26.7)** | **0(0)** | | **11(73.3)** | | | **7(8.23)** | | | **6(7.1)** | **72(84.7)** | | **0.049** |
| **Non-opiate pain medications take too long to work:** | **21(21)** | **33(33)** | **46(46)** | **9(60.0)** | **4(26.7)** | | **2(13.13)** | | | **12(14.5)** | | | **29(34.1)** | **44(51.7)** | | **< 0.001** |
| **Non-opiate pain medications aren't effective:** | **12(10)** | **12(12)** | **78(78)** | **6(40.0)** | **4(26.7)** | | **5(33.3)** | | | **4(4.7)** | | | **8(9.4)** | **73(85.9)** | | **< 0.001** |
| Giving Non-opiate pain medications will result in lower patient satisfaction: | 11(11) | 17(17) | 72(72) | 2(13.3) | 3(20.0) | | 10(66.7) | | | 9(10.6) | | | 14(16.5) | 62(72.9) | | 0.706 |
| ketamine should be used for pain management in the prehospital setting: | 72(72) | 19(19) | 7(7) | 12(80.0) | 2(13.3) | | 1(6.7) | | | 61(71.2) | | | 17(20.5) | 6(7.3) | | 0.88 |
| lidocaine nerve blocks should be used for pain management in the prehospital setting: | 33(33) | 38(38) | 29(29) | 3(20.0) | 7(46.7) | | 5(33.3) | | | 30(35.3) | | | 21(36.5) | 24(28.2) | | 0.339 |
| My service needs more specific protocols to determine which pain medication a patient receives: | 26(26) | 24(24) | 50(50) | 7(46.7) | 2(13.3) | | 6(40.0) | | | 19(22.3) | | | 22(25.9) | 44(51.7) | | 0.101 |

| **Supplemental Table 2.** Paramedic Perception Survey. | |
| --- | --- |
| Age |  |
| Gender | 1, Male \| 2, Female |
| Years As Practicing Paramedic |  |
| I have given fentanyl to a patient: | 1, Yes \| 2, No |
| I have given acetaminophen, ketorolac, or ibuprofen for pain in the prehospital setting | 1, Yes \| 2, No |
| I plan to give ibuprofen, acetaminophen or ketorolac to my patients in the future | 1, Yes \| 2, No |
| At what 'pain scale' number (1-10) do you give opiate pain medicine? | 1-10 |
| At what 'pain scale' number (1-10) would you give a non-opiate pain medication (Tylenol, acetaminophen or ketorolac) | 1-10 |
| What percentage of patients with a "pain complaint" do you give pain medication to? | 1, 0-25% \| 2, 26-50% \| 3, 51-75% \| 4, 76-100% |
| It is difficult to accurately gauge pain levels in the prehospital setting: | 1, Strongly Disagree \| 2, Disagree \| 3, Neutral \| 4, Agree \| 5, Strongly Agree |
| I do not give pain medication if the patient doesn't have an "obvious" medical problem/injury | 1, Strongly Disagree \| 2, Disagree \| 3, Neutral \| 4, Agree \| 5, Strongly Agree |
| I have a duty to provide pain relief in the prehospital setting | 1, Strongly Disagree \| 2, Disagree \| 3, Neutral \| 4, Agree \| 5, Strongly Agree |
| Pain relief in the prehospital setting is effective: | 1, Strongly Disagree \| 2, Disagree \| 3, Neutral \| 4, Agree \| 5, Strongly Agree |
| The goal of pain management in the prehospital setting is to eliminate all pain | 1, Strongly Disagree \| 2, Disagree \| 3, Neutral \| 4, Agree \| 5, Strongly Agree |
| The goal of pain management is the prehospital setting is to "take the edge off" | 1, Strongly Disagree \| 2, Disagree \| 3, Neutral \| 4, Agree \| 5, Strongly Agree |
| I hesitate to give pain medication to patients who I suspect have a history of opiate abuse because I am worried that they are "drug seeking" | 1, Strongly Disagree \| 2, Disagree \| 3, Neutral \| 4, Agree \| 5, Strongly Agree |
| I hesitate to give pain medications to patients who I suspect have a history of opiate abuse because I am worried I won't be able to overcome their tolerance to pain: | 1, Strongly Disagree \| 2, Disagree \| 3, Neutral \| 4, Agree \| 5, Strongly Agree |
| I hesitate to give pain medication to patients because I might change their clinical presentation for the emergency department | 1, Strongly Disagree \| 2, Disagree \| 3, Neutral \| 4, Agree \| 5, Strongly Agree |
| My transport time impacts my decision to give pain medicine: | 1, Strongly Disagree \| 2, Disagree \| 3, Neutral \| 4, Agree \| 5, Strongly Agree |
| I am hesitant to give opiate pain medicine because I am worried about the unwanted side effects: | 1, Strongly Disagree \| 2, Disagree \| 3, Neutral \| 4, Agree \| 5, Strongly Agree |
| I am hesitant to give non-opiate pain medicine because I am worried about the unwanted side effects: | 1, Strongly Disagree \| 2, Disagree \| 3, Neutral \| 4, Agree \| 5, Strongly Agree |
| Non-opiate pain medications take too long to work: | 1, Strongly Disagree \| 2, Disagree \| 3, Neutral \| 4, Agree \| 5, Strongly Agree |
| Non-opiate pain medications aren't effective: | 1, Strongly Disagree \| 2, Disagree \| 3, Neutral \| 4, Agree \| 5, Strongly Agree |
| Giving Non-opiate pain medications will result in lower patient satisfaction: | 1, Strongly Disagree \| 2, Disagree \| 3, Neutral \| 4, Agree \| 5, Strongly Agree |
| Ketamine should be used for pain management in the prehospital setting: | 1, Strongly Disagree \| 2, Disagree \| 3, Neutral \| 4, Agree \| 5, Strongly Agree |
| Lidocaine nerve blocks should be used for pain management in the prehospital setting: | 1, Strongly Disagree \| 2, Disagree \| 3, Neutral \| 4, Agree \| 5, Strongly Agree |
| My service needs more specific protocols to determine which pain medication a patient receives: | 1, Strongly Disagree \| 2, Disagree \| 3, Neutral \| 4, Agree \| 5, Strongly Agree |
